# Electrochemically Driven Hydrogen Atom Transfer Catalysis: A Tool for C(sp^3^)/Si–H Functionalization and Hydrofunctionalization of Alkenes

**DOI:** 10.1021/acscatal.3c01221

**Published:** 2023-06-16

**Authors:** Sheng Zhang, Michael Findlater

**Affiliations:** † Department of Chemistry and Biochemistry, University of California Merced, Merced, California 95343, United States; ‡ Institutes of Physical Science and Information Technology, Key Laboratory of Structure and Functional Regulation of Hybrid Materials of Ministry of Education, Anhui University, Hefei, Anhui 230601, China

**Keywords:** hydrogen atom transfer catalysis, electrochemical transformation, C(sp^3^)−H functionalization, hydrofunctionalization of alkenes

## Abstract

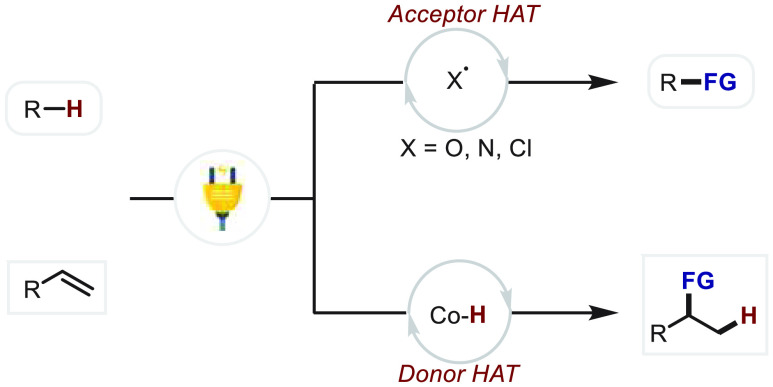

Electrochemically driven hydrogen atom transfer (HAT) catalysis provides a complementary approach for the transformation of redox-inactive substrates that would be inaccessible to conventional electron transfer (ET) catalysis. Moreover, electrochemically driven HAT catalysis could promote organic transformations with either hydrogen atom abstraction or donation as the key step. It provides a versatile and effective tool for the direct functionalization of C(sp^3^)–H/Si–H bonds and the hydrofunctionalization of alkenes. Despite these attractive properties, electrochemically driven HAT catalysis has been largely overlooked due to the lack of understanding of both the catalytic mechanism and how catalyst selection should occur. In this Review, we give an overview of the HAT catalysis applications in the direct C(sp^3^)–H/Si–H functionalization and hydrofunctionalization of alkenes. The mechanistic pathways, physical properties of the HAT mediators, and state-of-the-art examples are described and discussed.

## 1 Introduction

The term hydrogen atom transfer (HAT)^1^ is generally used to describe the concerted process which transfers a proton and an electron in the direction from a single donor to a single acceptor (Y^•^ + H–X → Y–H + X^•^). The HAT process is one of the subsets of proton-coupled electron transfer (PCET),^2−12^ which has been well-defined by Hammes-Schiffer as “any process that entails the combined movement of at least one electron and one proton”.^12^ Consequently, it should be noted that the HAT process is distinct from multiple-site concerted proton–electron transfer (MS-CPET);^1^ the process transfers a proton and an electron to/from spatially distinct sites or even different reagents.

The HAT process is known to be one of the elementary steps in alkane halogenation, which is often the first reaction taught in organic chemistry classes. The importance of the HAT step has been demonstrated in hydrocarbon combustion, atmospheric chemistry, and enzymatic catalysis^13^ over a century of research efforts. Specifically, in biology, the heme cytochrome P450 oxidizes a variety of organic molecules^14^ by use of a hydrogen atom abstraction step with the catalytically active center being a high-valent iron oxo species. Through synthetic mimicry of the biological processes, photoinduced HAT catalysis^15−18^ has emerged as a robust tool for the direct elaboration of C(sp^3^)–H bonds. As C(sp^3^)–H bonds are ubiquitous in petrochemicals and pharmaceuticals, selective functionalization of similar C–H bonds attracts enormous attention from both academia and the chemical industry, and it is regarded as one of the “Holy Grails” of modern synthetic chemistry.^19−25^ Photoinduced HAT catalysis is attractive for several reasons which are extensively reviewed elsewhere,^15−18^ but arguably the biggest advantage this approach represents is the ability to activate C(sp^3^)–H bonds without limitation of their redox potentials.

**Figure 1:**
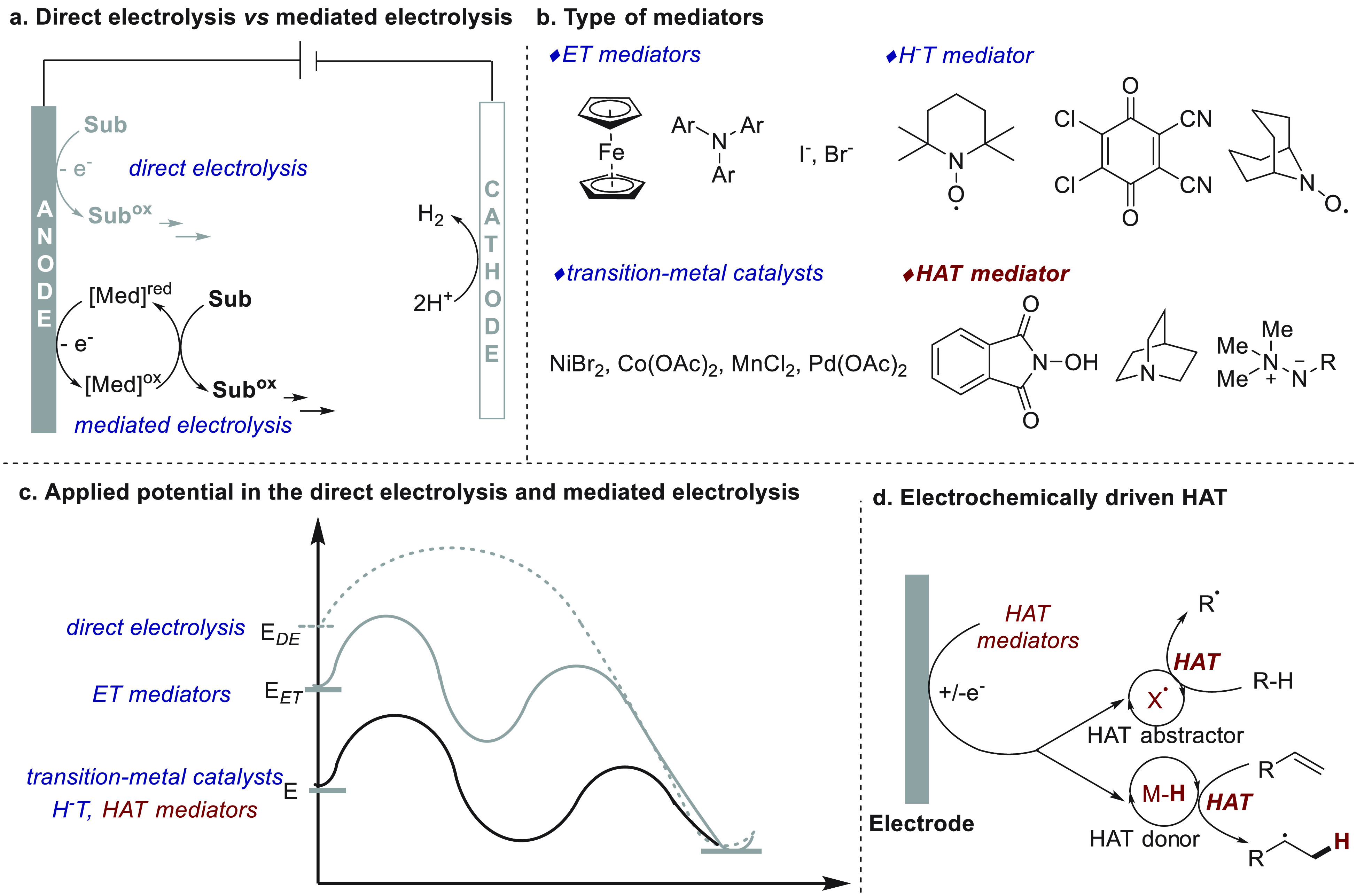
Comparison between direct and mediated electrolysis and the definition of electrochemically driven HAT.

Synthetic organic electrochemistry is another vehicle for redox chemistry, and it has experienced a renaissance^26−38^ in the past decade. With the introduction of redox mediators, the electrode will not directly participate in the chemical reaction, and the active catalyst is generated via the mediators to promote the reaction in the homogeneous solution. As a result, the electrochemical transformations can be accelerated at lower applied potential (vs. direct electrolysis in electrochemical oxidation, Figure 1a) obviating the overoxidation of products and the passivation of electrodes. Electron transfer (ET) mediators, such as ferrocene, triarylamine, and halogenide salts have been extensively explored in electrocatalysis and are well-reviewed^26^ (Figure 1b). As described in the review from Little and co-workers,^26^ a rule of thumb for ET mediators is the requirement of a narrow potential difference (<0.5 V) with the starting materials. This limitation makes ET mediators incompatible with redox-inactive substrates. To broaden the reaction scope and compatibility, other modes of electrocatalysis have been developed, including transition-metal catalysts, hydride transfer (H-T) mediators, and HAT mediators (Figure 1b). The driving force of the catalytic process is based on the chemical reactivity rather than the potential difference between the mediator and substrate; thus, applied potential can be significantly reduced even by >1.0 V (Figure 1c). The merger of transition-metal catalysis and electrooxidation (or -reduction) has paved a novel pathway for C–H activation and cross-coupling reactions, and the recent progress in the field has been reviewed by Ackermann,^39^ Mei,^40^ and Minteer.^41^ Impressive achievements in the electrochemical oxidation with H-T mediators have been reported and summarized by the Stahl group.^42^ In sharp contrast, the development of electrochemically driven HAT catalysis continues to be sluggish. Although the robustness of electrochemical PCET in theoretical study, energy conversion, and organic synthesis has been widely demonstrated and summarized by Hammes-Schiffer^12^ and Knowles,^11^ the PCET process reported in the review mainly involves the MS-CPET or sequential proton/electron transfer. Electrochemically driven HAT can be defined as the process using an electrochemically generated HAT abstractor or a donor to initiate the hydrogen atom transfer with substrates (Figure 1d). This chemistry enables appealing approaches for the direct functionalization^43^ of C(sp^3^)–H bonds and the hydrofunctionalization of alkenes. Despite these promising applications, its utility has been largely overlooked, perhaps because of the continued poor understanding of catalytic mechanisms and catalyst selection.

Hence, it would be timely to compile the most recent developments in electrochemically driven HAT catalysis. This Review aims to provide guidance for the application of HAT catalysis in direct C(sp^3^)/Si–H functionalization and hydrofunctionalization of alkenes. Mechanisms of electrochemically driven HAT catalysis (in solution), factors (bond dissociation energy, redox potential) affecting HAT catalysis, and state-of-the-art examples of the aforementioned transformations are included in this Review. The detailed mechanism over the surface of the electrode will fall outside the scope of this Review for the sake of brevity.

## 2 The Electrochemically Driven HAT Mechanism and Differences between the Photoinduced HAT Mechanism

**Figure 2:**
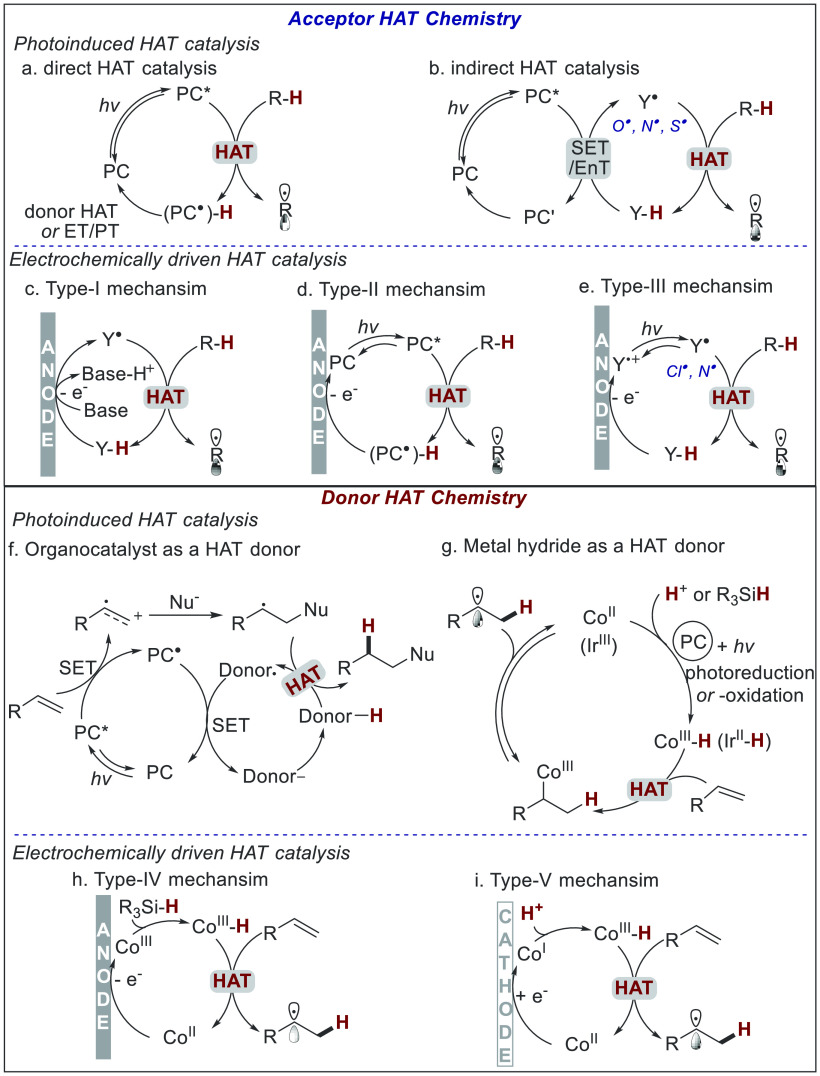
Mechanisms of photoinduced HAT catalysis and electrochemically driven HAT catalysis.

To better understand the electrochemically driven HAT catalysis mechanism, we will begin with a brief description of the more well elaborated photoinduced HAT catalytic cycles and draw comparisons between these related processes (Figure 2). In general, HAT catalysis may promote organic transformations with either hydrogen atom abstraction or donation as the key step. These two mechanisms can be described as acceptor and donor HAT chemistry, respectively. The electrochemically driven HAT catalysis is compatible with both acceptor and donor HAT chemistry. Additionally, through the combination of electrochemistry and photoredox catalysis, the mechanisms accessible during electrochemically driven HAT catalysis have been diversified.

For the acceptor HAT chemistry, the photoinduced mechanism typically occurs through two pathways, direct and indirect HAT catalysis.^15,16^ In the case of direct HAT catalysis (Figure 2a), photocatalyst (PC) is converted to an excited triplet state (PC*) which may directly abstract hydrogen atom from R–H to generate alkyl radical (R^•^) and (PC^•^)–H. The catalyst (PC) can be regenerated from (PC^•^)–H via hydrogen atom donation or sequential steps of ET and proton transfer (PT). The indirect HAT catalysis mode (Figure 2b) relies on the synergistic interplay between PC and cocatalyst (Y–H). Heteroatom-centered radicals (Y^•^, e.g., O^•^, S^•^, N^•^) are generated *in situ* from cocatalyst (Y–H) with concomitant activation of excited photocatalyst (PC*) through a single electron transfer (SET) or energy transfer (EnT) process. The heteroatom-centered radicals (Y^•^) are capable of abstracting hydrogen atoms from various R–H bonds to give alkyl radical (R^•^) and regenerate cocatalyst Y–H.

On the other hand, the electrochemical acceptor HAT chemistry can proceed using three types of pathways. The first type is directly driven by electricity^44−47^ (named as type-I mechanism, Figure 2c), in which the electrochemically generated heteroatom-centered radicals or radical cations (Y^•^, e.g., O^•^, N^•^, N^+•^) abstract a hydrogen atom from R–H to deliver active alkyl radical R^•^ and regenerate catalyst (Y–H). This step requires the precursors of the heteroatom-centered radical (Y–H) to be more susceptible to anodic oxidation than the substrates (R–H). Moreover, the bond dissociation energy of Y–H should be much higher than that of R–H to serve as the driving force of the HAT process. Upon light excitation, photoelectrochemical HAT catalysis can proceed via type-II or type-III mechanisms. In the type-II mechanism (Figure 2d), the excited photocatalyst (PC*) abstracts hydrogen atoms from R–H to give alkyl radicals (R^•^), and this process is closely related to the direct photoinduced HAT.^48,49^ The major difference is that the photocatalyst (PC) is regenerated via anodic oxidation of (PC^•^)–H. The type-III mechanism (Figure 2e) generates a hydrogen atom abstractor (Cl^•^ or N^+•^) through sequential anodic oxidation and photoexcitation,^50,51^ and the hydrogen atom abstractor then triggers the HAT process.

The donor HAT chemistry has been widely explored in the photocatalyzed hydrofunctionalization of alkenes.^52−56^ Mechanistically, photoinduced HAT donation can be accessed by using an organocatalyst or metal-hydride as a HAT donor (Figure 2f,g). The mechanism involving organic HAT donors has been well concluded by Nicewicz and co-workers (Figure 2f).^52^ In the catalytic cycle, the hydrogen atom donation process serves as a radical termination step in the hydrofunctionalization of alkene, and the HAT donor was regenerated through photoreduction and protonation. In contrast, the metal-hydride mechanism generates Co(III)–H (or Ir(II)–H) as the reactive HAT donor via photoreduction or photooxidation, and the HAT donor initiates the hydrofunctionalization of alkenes by generating a carbon-centered radical (Figure 2g).^53−56^

Electrochemically driven HAT catalysis also provides alternative pathways for donor HAT chemistry. As described in recent reports, this catalytic mode can proceed using either anodic (type-IV) or cathodic (type-V) pathways and employing different hydrogen atom sources (hydride and protons). Under anodic oxidation (Figure 2h), a Co(II) catalyst is oxidized to a Co(III) species,^57,58^ which reacts with silanes to give a metal-hydride Co(III)–H. Co(III)–H can act as a hydrogen atom donor via homolytic cleavage to transfer a hydrogen atom to an alkene and regenerate the Co(II) catalyst. In contrast, the cathodically driven donor HAT catalysis (Figure 2i)^59,60^ begins with the cathodic reduction of a Co(II) catalyst, and the resulting low-valent Co(I) species reacts with proton (H^+^) to deliver Co(III)–H, that undergoes similar HAT steps to close the catalytic cycle.

Comparison between the photoinduced HAT with the electrochemically driven HAT catalysis reveals that the electrochemical protocol seems to be simpler and more tunable,^38^ as it commonly obviates the usage of photoredox catalysts, and the redox potential in the reaction is continuously adjustable. Consequently, the electrochemically driven HAT catalysis may serve as a complementary approach for the photoinduced protocol.

## 3 Classification of HAT Mediators and Their Physical Properties

**Scheme 1:**
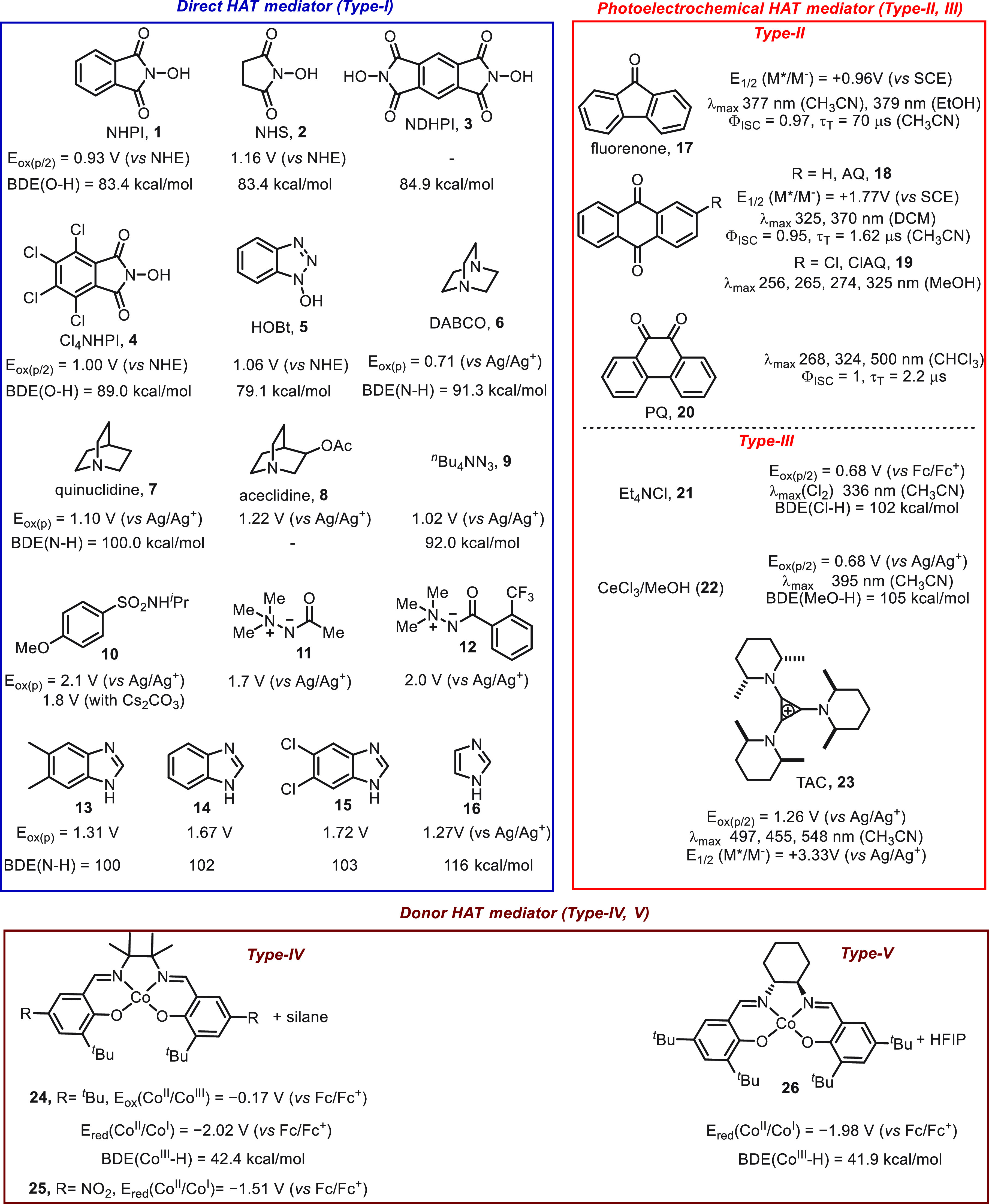
Physical Properties of the HAT Mediators Described in This Review

To help researchers make informed decisions on the selection of appropriate HAT mediators, we have summarized the types of electrochemical mediators and their physical properties^16,17,42,45−47,51,58−70^ (Scheme 1) which directly affect their catalytic performance. Based upon our description of electrochemically driven HAT mechanisms, we have classified HAT mediators into three types, direct HAT mediator, photoelectrochemical HAT mediator, and donor HAT mediator. As mentioned above, the driving force of the acceptor HAT mediator (including direct HAT mediator, and photoelectrochemical HAT mediator) relies upon their lower oxidation potential and higher bond dissociation energy (BDE) compared to the substrates (R–H), although the lifetime of the heteroatom-centered radicals formed in the reaction, as well as the UV–vis absorption properties of photoelectrochemical HAT mediators, is also critical to their catalytic efficiency. The donor HAT mediators are also required to have lower oxidation potential for the anodic HAT pathway (or more positive reduction potential for the cathodic HAT pathway), and the hydrogen atom transfer to an alkene substrate should be extremely efficient to outcompete the hydrogen evolution reaction (HER) which is a competing reaction pathway.

From the standpoint of catalyst design, the catalytic performance can be improved through structural modification. For instance, the introduction of a large conjugated system and electron-withdrawing groups into the direct HAT (Type-I) mediator may serve as solutions^70^ to lower the anodic potential and strengthen the BDE, respectively. Increasing the steric hindrance of the mediator is also helpful for the catalyst stability and efficiency, as the undesired dimerization of radicals can be suppressed. In the case of a photoelectrochemical HAT mediator, the catalytic performance is governed by multiple factors, and the catalyst design can partially learn from the review of the photoinduced HAT.^15,16^ The modification of the donor HAT mediator can be achieved by varying metal centers (Fe, Ni, Cu, etc.) or ligands. As demonstrated in the recent report of Baran,^59^ ligands significantly affected the BDE of Co(III)–H and Co(II)–C bonds. The weak Co(III)–H and Co(II)–C bonds of cobalt salen proved to be beneficial for the radical type of HAT pathway.

Prior to discussing electrochemically driven HAT catalysis, nomenclature related to interpreting reaction conditions is presented to help readers more fully understand them. The electrode materials are placed over the arrows, and the polarity of the electrode is denoted as a (+) sign for the anode and a (−) sign for the cathode. For example, “C(+)–Pt(−)” indicates that electrolysis uses a graphite anode and a platinum cathode. Current is provided for the electrolysis performed under constant current electrolysis (CCE); an anode potential (*E*_a_) is provided for constant potential electrolysis (CPE) and cell potential (*E*_cell_), for constant voltage electrolysis.

## 4 HAT Catalysis in the Electrochemical Functionalization of C(sp^3^)/Si–H

The electrochemical functionalization of the C(sp^3^)–H bond, which is enabled by HAT catalysis, begins with the acceptor HAT process to generate an open-shell R^•^ species. The direct HAT and photoelectrochemical HAT mediators are typically involved in these transformations. Thus, we will describe progress in this area by classification of transformations into categories according to the mediator responsible for HAT catalysis.

### 4.1 C(sp^3^)–H Functionalization by Direct HAT Mediator

**Scheme 2:**
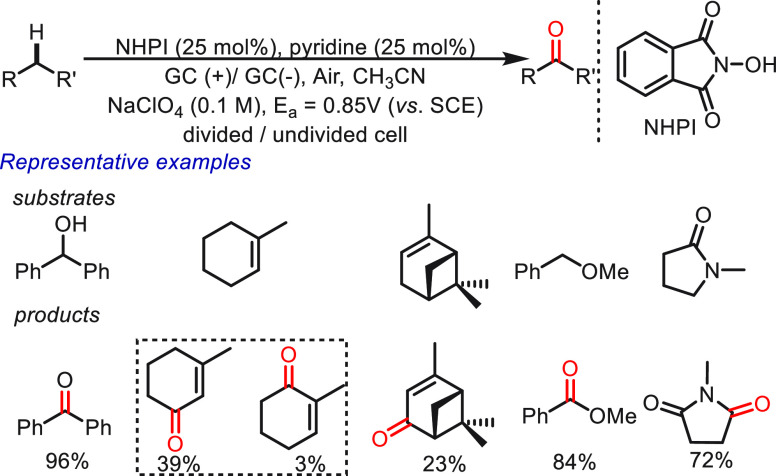
Electrochemical C–H Oxidation Mediated by NHPI

Although the HAT step has been long known as an elementary step in the halogenation of alkanes, the electrochemical application of HAT chemistry received attention until the 1980s. In 1977, Grochowski^71^ reported the first free radical reaction catalyzed by *N*-hydroxyphthalimide (NHPI) in the presence of external oxidants. Inspired by the seminal work, NHPI was first explored by Masui and co-workers^72−74^ as a direct HAT mediator in the anodic oxidation of C(sp^3^)–H bonds using molecular oxygen (Scheme 2). In the HAT protocol, a range of molecules, such as alcohols, amides, allylic, and benzylic substrates, were converted to oxidation products. Nevertheless, the electrochemical oxygenation of allylic C(sp^3^)–H bonds still suffered from inferior site-selectivity and low yield owing to the restriction of electrode materials at that time.

**Scheme 3:**
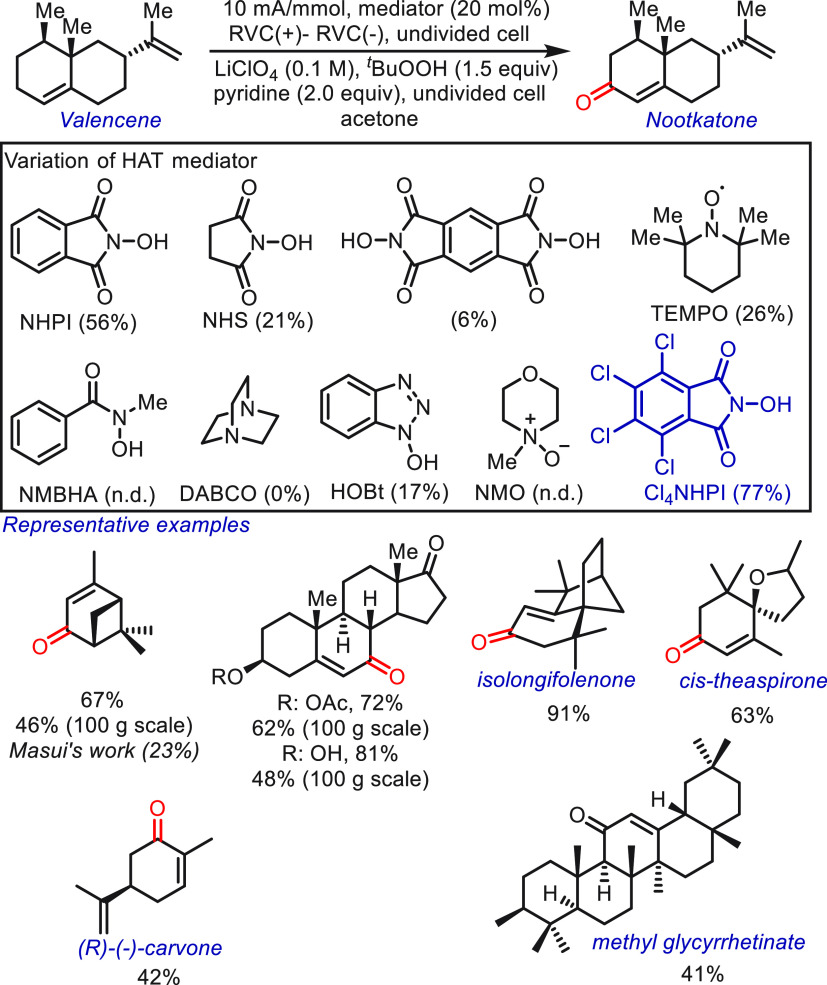
Electrochemical Allylic C–H Oxidation Mediated by Cl_4_NHPI

With increasing concerns about sustainable chemistry, tremendous effort has been devoted to synthetic organic electrochemistry in the 21st century. The electrochemically driven HAT catalysis also revives with the promotion of the theoretical study in the HAT^1−10^ and the related study on NHPI.^75,76^ In 2016, Baran^44^ and co-workers reported a solution to address the aforementioned issues in the oxygenation of allylic C(sp^3^)–H (Scheme 3). The methodological improvements include the addition of a simple co-oxidant (*t*-butyl hydroperoxide, TBHP), using more reactive NHPI derivatives (Cl_4_NHPI), and an anode material (reticulated vitreous carbon, RVC) that was previously less explored in organic electrochemistry. The results obtained using different HAT mediators are presented to help facilitate an understanding of the structure-performance relationships. A more electron-deficient mediator, Cl_4_NHPI, has a stronger O–H bond, which is beneficial for the hydrogen atom abstraction. Under optimized reaction conditions, the efficiency and selectivity of the transformation were significantly improved, and a broad range of substrates (>30) was amenable to afford structurally complex natural products. As demonstrated in the reaction of α-pinene, the yield of product verbenone was enhanced from 23% to 67%. Some impressive products, such as isolongifolenone, cistheaspirone, (*R*)-(−)-carvone, and methyl glycyrrhetinate, are also highlighted. Notably, this HAT protocol showed excellent compatibility for larger scale transformations (100 g scale reactions) with less-hazardous electrolyte LiBF_4_ and inexpensive graphite electrodes. This electrochemical protocol provides a selective approach to accessing allylic C(sp^3^)–H oxidation, although the usage of peroxide partially restricts the practical application.

**Figure 3:**
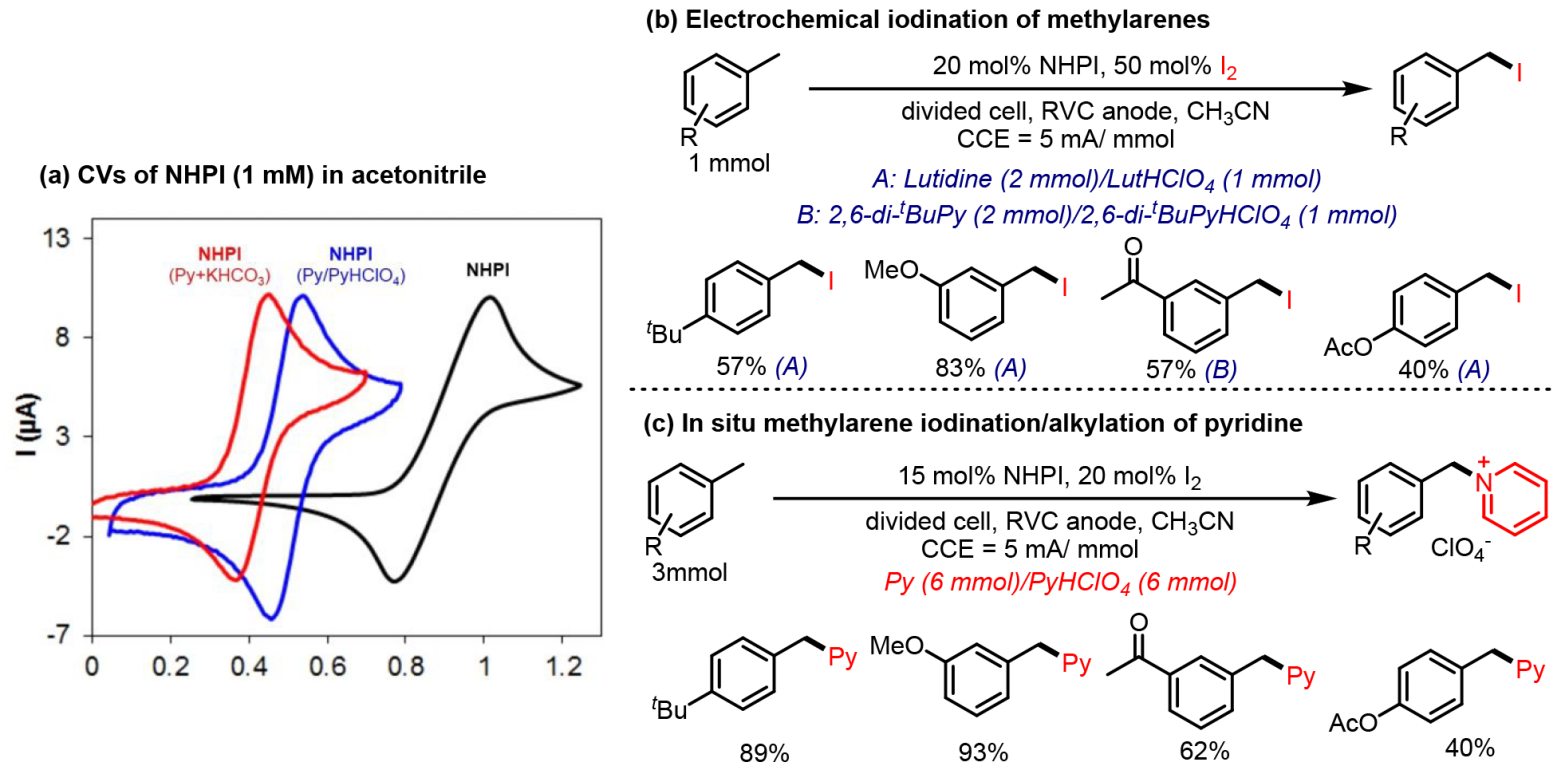
Electrochemical iodination of methylarenes mediated by NHPI.

To further extend the utility of direct HAT catalysis in the functionalization of C(sp^3^)–H bonds, heteroatom reagents other than oxygen were introduced to trap the alkyl radicals generated *in situ*. In 2018, Stahl^77^ reported a novel approach to the iodination of methyl arenes with molecular iodine as a trapping reagent (Figure 3). It was found that a buffered solution of pyridine/pyridinium can improve the stability of O-centered radical phthalimido-*N*-oxyl (PINO) when compared with the use of pyridine and inorganic base, and the highest cathodic-to-anodic peak current ratio (*I*_c_/*I*_a_ = 0.96) was observed (Figure 3a). After screening reaction conditions, sterically hindered pyridine analogs (Lutidine or 2,6-di-^*t*^BuPy) were shown to afford the desired iodination product with moderate to good yields (Figure 3b). Interestingly, increasingly nucleophilic pyridine/pyridinium electrolytes led to the observation of benzylpyridinium products via a sequential nucleophilic substitution with benzylic iodine products (Figure 3c).

**Scheme 4:**
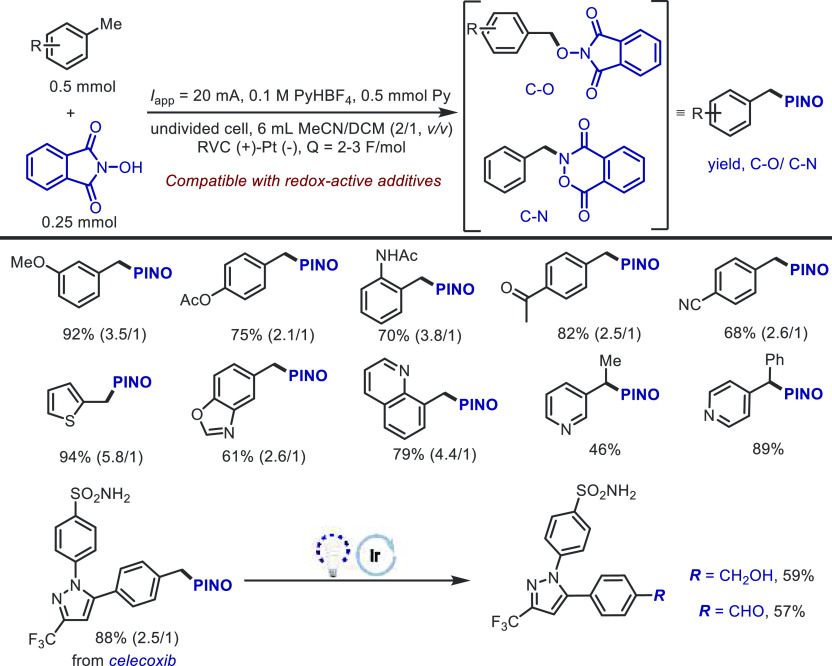
Electrochemical PINOylation of Methylarenes

The same group^78^ recently disclosed that electrochemically generated PINO could serve dual roles of HAT mediator and trapping reagent for alkyl radicals (Scheme 4). Although a mixture of amination and oxygenation products was observed, which exhibited only moderate selectivity, this protocol revealed unprecedented functional group tolerance; carbonyl, nitrile, amide, ester, and heterocycles are all well-tolerated. The robustness of the HAT protocol was highlighted by the comparison with the direct oxidation of methylarenes and the compatibility with external additives. A wide range of redox-active additives was readily recovered due to the significantly lowered applied potential in the reaction. Additionally, the synthetic utility of this protocol was demonstrated by the photochemical diversification of products and the late-stage derivatization of celecoxib.

**Scheme 5:**
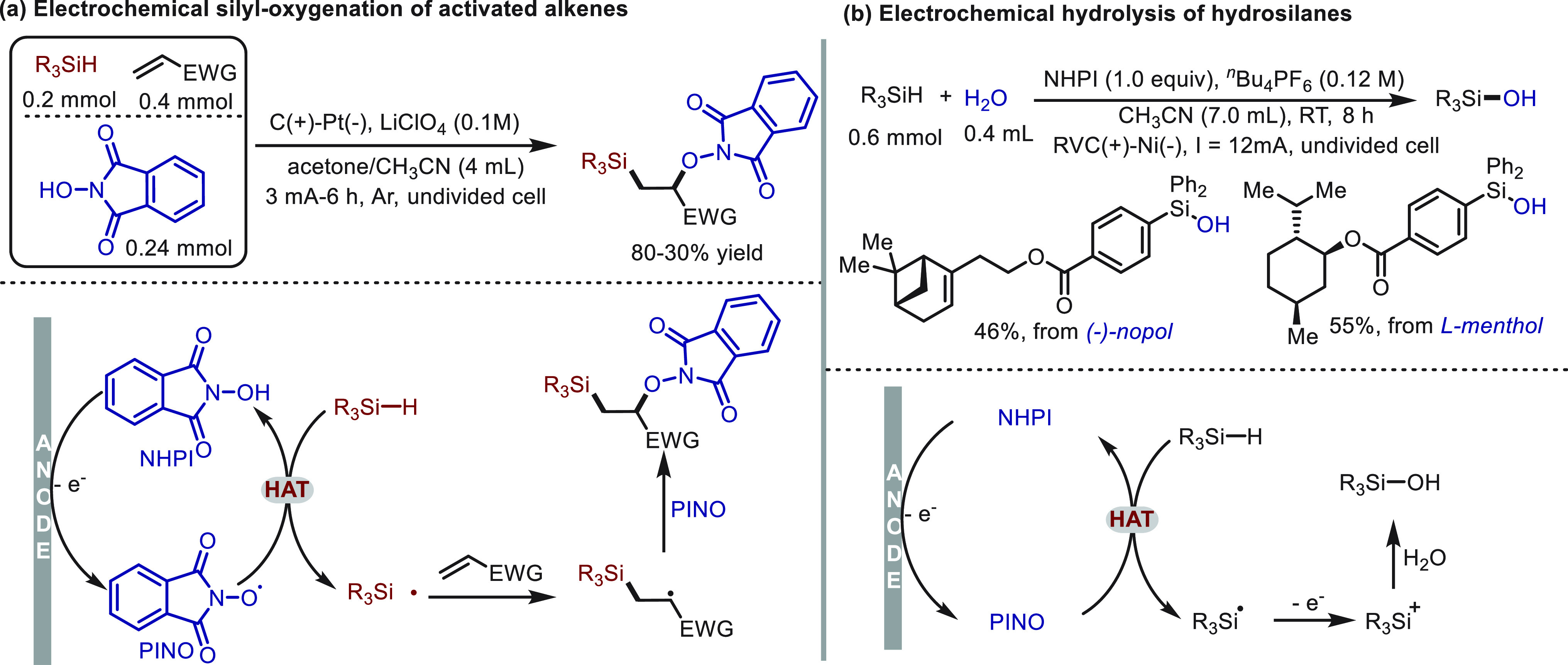
Electrochemical Activation of Si–H Bonds Mediated by NHPI

The catalytic versatility of NHPI was independently demonstrated by the Zhang^79^ and He^80^ groups in the activation of Si–H bonds, which have a close relationship to C(sp^3^)–H bonds (Scheme 5). Using a HAT catalysis strategy, organosilane (*E*_ox_ ∼ 2.0 V vs. Ag/Ag^+^) was oxidized to silyl radical, which could be directly intercepted by electron-poor alkenes and proceed through subsequent radical coupling with PINO to afford silyl-oxygenation products (Scheme 5a). Alternatively, the silyl radical undergoes further SET oxidation at the anode to deliver a silyl cation. In the presence of water, hydrolysis products were readily accessed (Scheme 5b). Some representative products arising from natural products are presented.

**Scheme 6:**
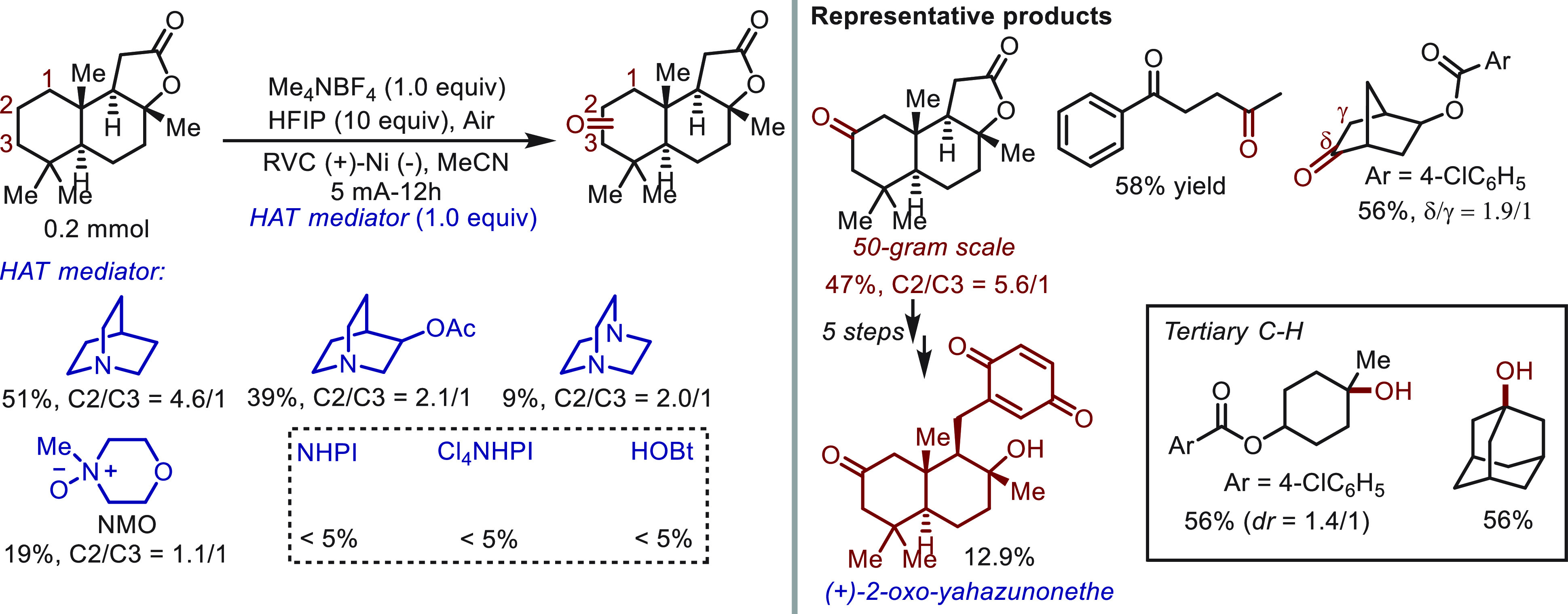
Electrochemical Oxidation of Nonactivated C–H Bonds Mediated by Quinuclidine

The direct HAT mediators based on nitrogen-centered radicals were also evaluated by Baran^45^ in the direct oxygenation of C(sp^3^)–H bonds (Scheme 6). Despite their earlier work in the oxidation of allylic C(sp^3^)–H bonds, the oxidation of nonactivated methylene and methine groups received far less attention likely arising from their high anodic potential (>3.0 V vs. SCE), which was even higher than that of solvents. To address this challenging issue, quinuclidine was introduced in the electrochemical oxidation of sclareolide. After comparison with other HAT mediators, quinuclidine was found to be the optimal choice in terms of reaction efficiency and site-selectivity. The generality of the quinuclidine-mediated oxidation was demonstrated using a broad range of complex molecules and natural products. In addition to methylene groups, this protocol also worked well with methine groups to afford tertiary alcohol products. The practical utility of the approach was explored in the total synthesis of (+)-2-oxo-yahazunonethe by carrying out the large-scale C–H oxidation of sclareolide as the critical step.

**Scheme 7:**
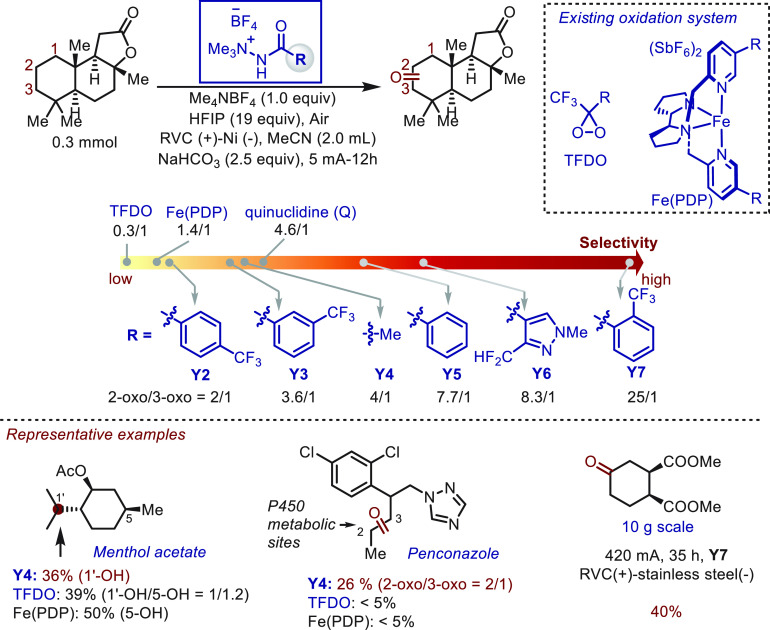
Electrochemical Oxidation of C–H Bonds Mediated by *N*-Ammonium Ylides

Three years later, the same group^46^ devised a novel class of *N*-ammonium ylides (80 analogs) as tunable HAT mediators (Scheme 7) that are rapidly accessed in two synthetic steps. The superior performance of the novel mediators was highlighted in the oxidation of sclareolide, and the highest site-selectivity (25/1) yet reported was achieved. This performance surpassed that of previously reported oxidation systems TFDO, Fe(PDP), and HAT mediator quinuclidine. Moreover, the distinctive site-selectivity of the novel *N*-ammonium ylides was observed in the oxidation of menthol acetate to give a single product. The synthetic utility of the HAT platform was also demonstrated in the “electrochemical metabolism” of Penconzaole. The propyl group in the molecule was successfully oxidized to the ketone product in 26% yield with a 2/1 ratio, while other known oxidation protocols failed to offer synthetically useful yields. The scalability of the approach was successfully demonstrated using the oxidation of dimethyl cyclohexane-1,2-dicarboxylate on a 10 g scale. In the preparative-scale reaction, a less expensive stainless-steel cathode was used in place of the nickel plate. This promising result suggests that HAT mediators based on nitrogen-centered radicals are also potent tools in electrochemical C–H functionalization chemistry.

**Figure 4:**
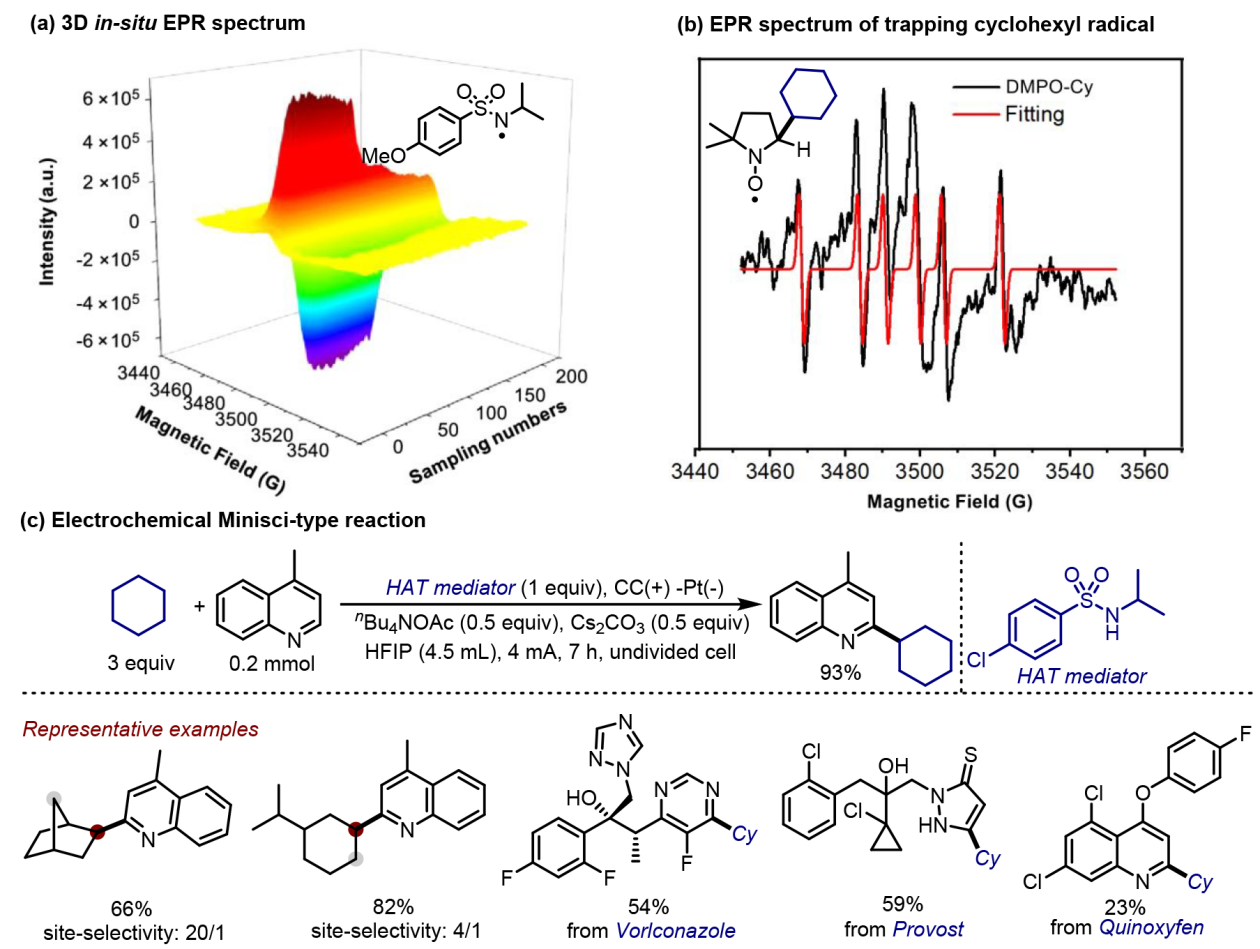
Electrochemical Minisci-type reaction mediated by sulfonamides.

In late 2021, Lei^47^ and co-workers reported an *in situ* electron paramagnetic resonance (EPR) technique for the detection of *N*-centered radicals arising from the anodic oxidation of sulfonamides (Figure 4). After unambiguously identifying the radical species (Figure 4a), its ability to abstract a hydrogen atom from cyclohexane was further confirmed via the detection of an EPR signal assigned to a cyclohexyl radical (adducts with 5,5-dimethyl-1-pyrroline-*N*-oxide, DMPO, Figure 4b). Given this appealing reactivity profile, a sulfonamide-mediated Minisci-type reaction was conducted; excellent functional group tolerance was observed, and a general approach to access heteroarylation products was developed (Figure 4c). Notably, promising site-selectivity was observed in the reaction of substrates bearing multiple potential sites of reactivity. The HAT approach allowed the direct functionalization of both pharmaceutical (Voriconazole) and agricultural chemicals (Provost and Quinoxyfen).

**Scheme 8:**
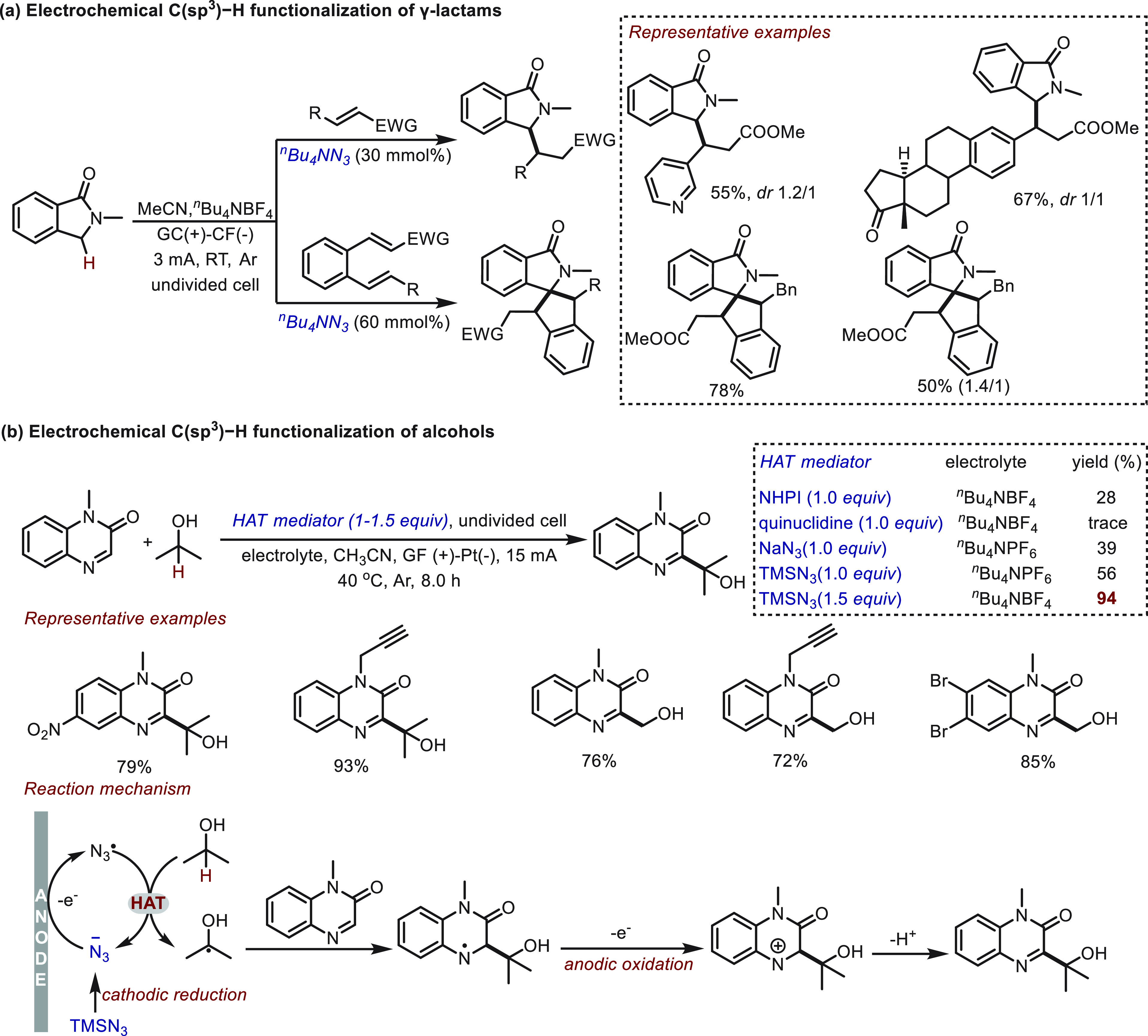
Electrochemical C–H Functionalization Mediated by Azide Mediator

Recently, azide salts were reported as alternative HAT mediators in the direct C(sp^3^)–H functionalization of γ-lactams and alcohols. Park and co-workers^81^ used tetrabutylammonium azide (^*n*^Bu_4_NN_3_) as a HAT mediator in the electrochemical reaction between γ-lactams and electron-deficient alkenes (Scheme 8a). An excellent functional group tolerance was readily achieved in both inter- and intramolecular reactions. Additionally, 1,2-divinyl substituted arenes were found to be suitable acceptors and capable of undergoing “double HAT” processes to afford spirocyclic products, albeit with increased catalyst loading (60 mol %). In the electrochemical Minisci reaction of alcohols, Sun and Liu^68^ discovered that TMSN_3_ served as a HAT mediator (Scheme 8b). When compared with other HAT mediators (NHPI, quinuclidine, NaN_3_), TMSN_3_ proved to be the most efficient, and desired product was delivered in excellent (94%) yield. Although a high loading (1.5 equiv) of TMSN_3_ was required in the transformation, a broad range of quinoxalinones and aliphatic alcohols was well tolerated. Specifically, methanol (which is a readily available C1 feedstock) was a suitable substrate to deliver hydroxymethylation products upon increasing to 2 equiv of TMSN_3_. Mechanistically, this electrochemical Minisci reaction proceeds via azide anion cathodic generation from TMSN_3_, which was followed by anodic oxidation to give an azide radical as the HAT species.

**Scheme 9:**
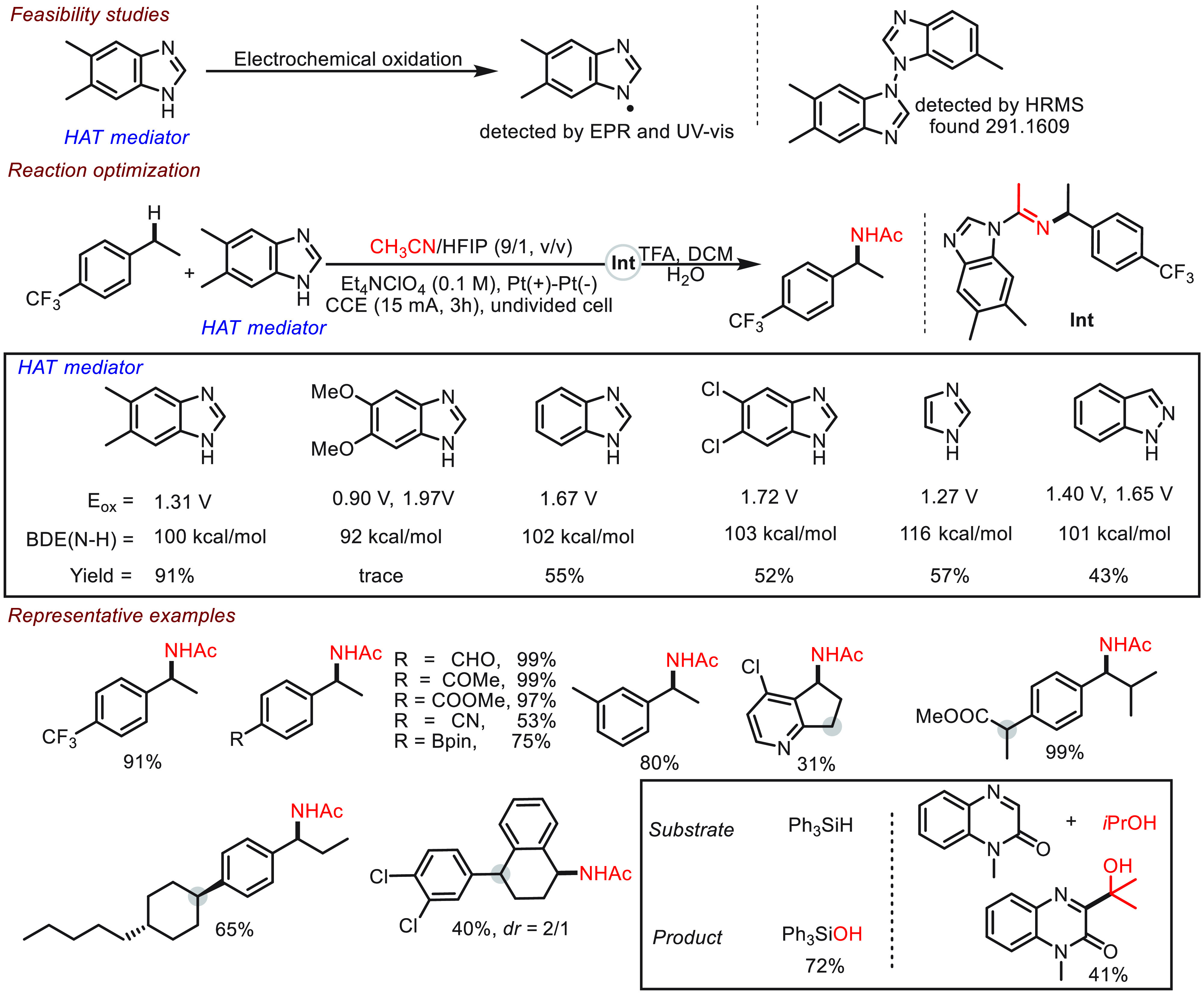
Electrochemical C(sp^3^)–H Amination Enabled by Benzimidazole Mediators

Very recently, Zhang and Li unveiled that benzimidazole variants could serve as efficient HAT mediators in the electrochemical Ritter-type amination of benzylic C(sp^3^)–H bonds^70^ (Scheme 9). To explore the feasibility of using benzimidazole as a HAT mediator, the nitrogen-centered radicals generated from benzimidazole variants were unambiguously identified through a series of analysis tools. To better understand the catalytic performance of benzimidazole in electrochemical amination, these authors listed oxidation potentials and the BDE of N–H bonds. As demonstrated in the work, low oxidation potential and high BDE (N–H) are necessities for high HAT activity. The superiority of the HAT mediator was also highlighted by the good tolerance for strongly deficient substrates, which are less explored in the previous reports. More interestingly, the HAT mediator exhibited unconventional site-selectivity as demonstrated in the representative examples. The catalytic generality of the HAT mediator was also demonstrated in the electrochemical Minisci-type reaction and silane oxidation.

### 4.2 C(sp^3^)–H Functionalization by Direct Photoelectrochemical HAT (Type II) Mediator

**Scheme 10:**
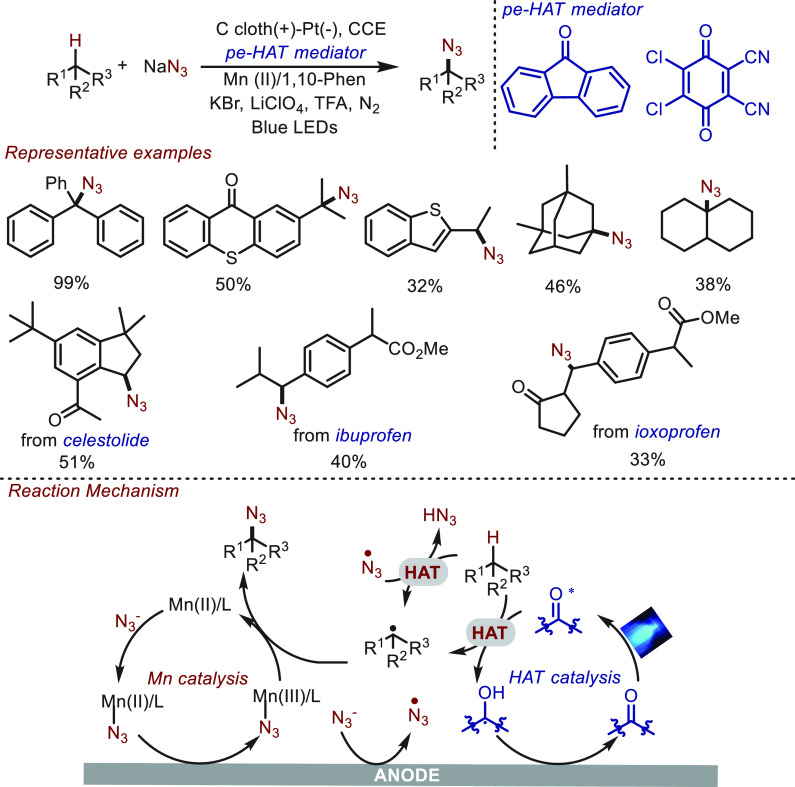
Photoelectrochemical C(sp^3^)–H Azidation

With the recent attention paid to both photoredox catalysis and synthetic electrochemistry, it is unsurprising that the combination of these two technologies provides an appealing strategy^82,83^ for carrying out transformations that would be inaccessible for either approach alone. The direct azidation of the C(sp^3^)–H bond is regarded as one such transformation, which conventionally involves excess oxidant or hypervalent iodine reagents. In 2020, Lei^84^ and co-workers reported an electrophotocatalytic approach for this challenging transformation by using photoelectrochemical HAT (type-II) mediators (Scheme 10). In this transformation, a series of tertiary and secondary benzylic C(sp^3^)–H, aliphatic C(sp^3^)–H, and pharmaceutical derivatives were readily azidated in moderate to excellent yields. Since this catalytic system consists of metal-based catalysis, electrochemistry, and photochemistry, the reaction mechanism deviates slightly from that described above (in Figure 2d). As depicted in Scheme 10, this reaction can be initiated with a HAT photocatalyst under the irritation of blue light. Alternatively, the HAT process could be triggered by anodically generated azide radicals. After transferring a hydrogen atom to azide radical or PC, an active alkyl radical was generated, which undergoes a subsequent azidation via manganese catalysis.

**Scheme 11:**
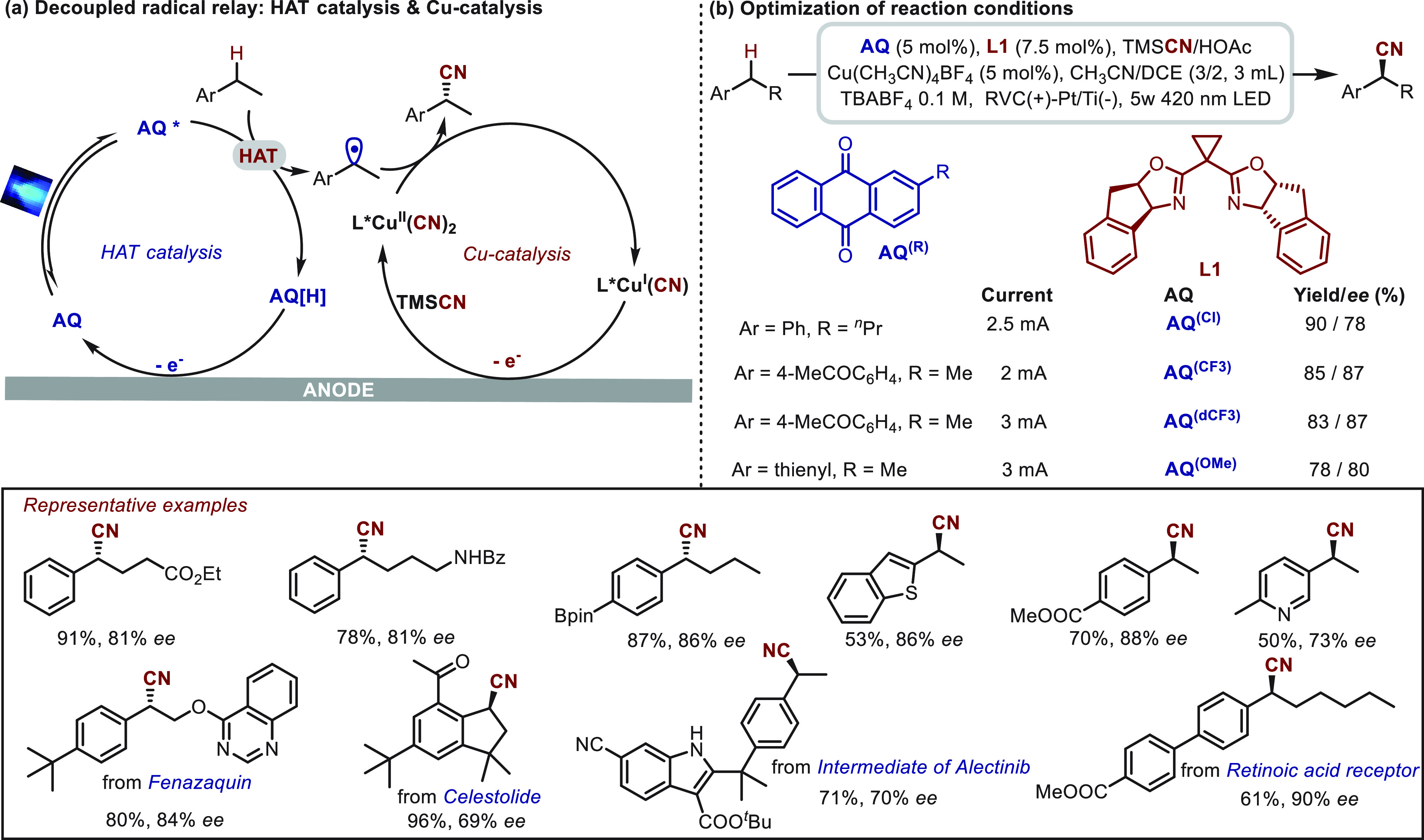
Enantioselective Benzylic C–H Cyanation Enabled by Photoelectrochemical HAT Catalysis

In 2022, an impressive work in the enantioselective benzylic C(sp^3^)–H cyanation was reported independently by the Xu^85^ and Liu^48^ groups, using a photoelectrochemical strategy. The successful application of this strategy to this transformation was ascribed to the catalytic mode of the decoupled radical relay. In Liu’s work, a tandem HAT process and copper oxidation was proposed for the decoupled radical relay (Scheme 11a). These two steps could be independently tuned by varying the electronic properties of an anthraquinone-type photocatalyst and modulating the applied current, thus substantially improving the functional-group compatibility (Scheme 11b). The optimization results for substrates with different electronic properties are described to help understand the tunability of the reaction. It should be noted that the complex structures derived from bioactive molecules were amenable to giving corresponding products with good yields (61–96%) and enantioselectivities (69–90%).

**Scheme 12:**
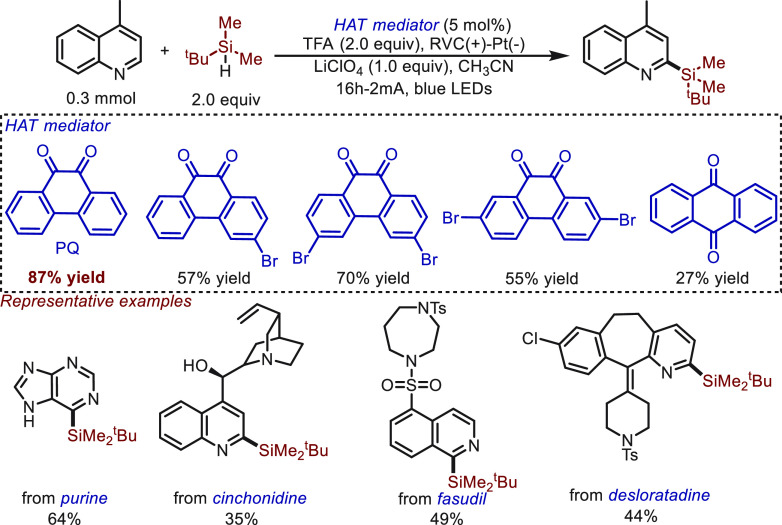
Electrochemical Minisci-Type Silylation Enabled by HAT Catalysis

Very recently, 9,10-phenanthrenequinone (PQ) was reported as a direct photoelectrochemical HAT mediator by Wang and Hou^49^ (Scheme 12). Using this novel mediator, a Minisci-type silylation reaction was readily achieved through the canonical mechanism of direct photoelectrochemical HAT (type II mechanism). Variation of the mediators showed that PQ was required for the transformation to be highly efficient. The reaction compatibility and utility were further demonstrated by the direct silylation of natural products and pharmaceuticals, including purine, cinchonidine, fasudil, and desloratadine.

### 4.3 C(sp^3^)–H Functionalization by Indirect Photoelectrochemical HAT (Type-III) Mediator

**Scheme 13:**
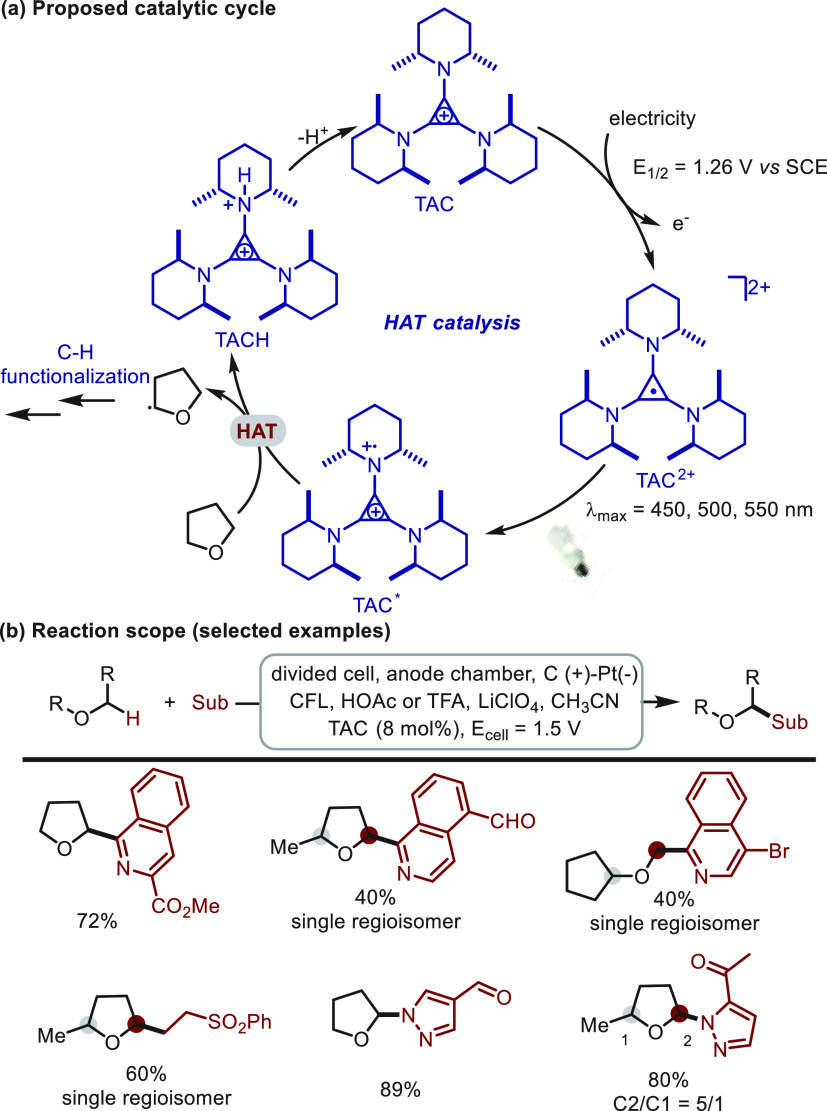
Electrochemical C(sp^3^)–H Functionalization of Ethers Mediated by TAC

Indirect photoelectrochemical HAT catalysis commonly generates a photoactive species via anodic oxidation, and the species further transforms into a reactive HAT catalyst under the irritation of light. In 2020, a rationally designed trisaminocyclopropenium (TAC) ion was reported by Lambert^50^ as an indirect photoelectrochemical HAT mediator (Scheme 13). In the photoelectrochemical transformation, the trisaminocyclopropenium (TAC) ion was oxidized to an air-stable radical dication, which was further excited under light irritation with long wavelength absorption (Scheme 13a). The excited species (TAC*) was proposed to abstract hydrogen atoms from ethers and facilitate a series of C(sp^3^)–H bond functionalizations at a very low cell potential (1.5–2.0 V). This indirect HAT catalysis strategy provided a mild platform featuring an excellent functional group (e.g., aldehyde, ketone, ester) tolerance (Scheme 13b). Moreover, site-selectivity was also observed for those ether substrates bearing multiple C(sp^3^)–H bonds. This selectivity was ascribed to the sterically hindered nature of TAC radical dication and stability of carbon radicals.

**Scheme 14:**
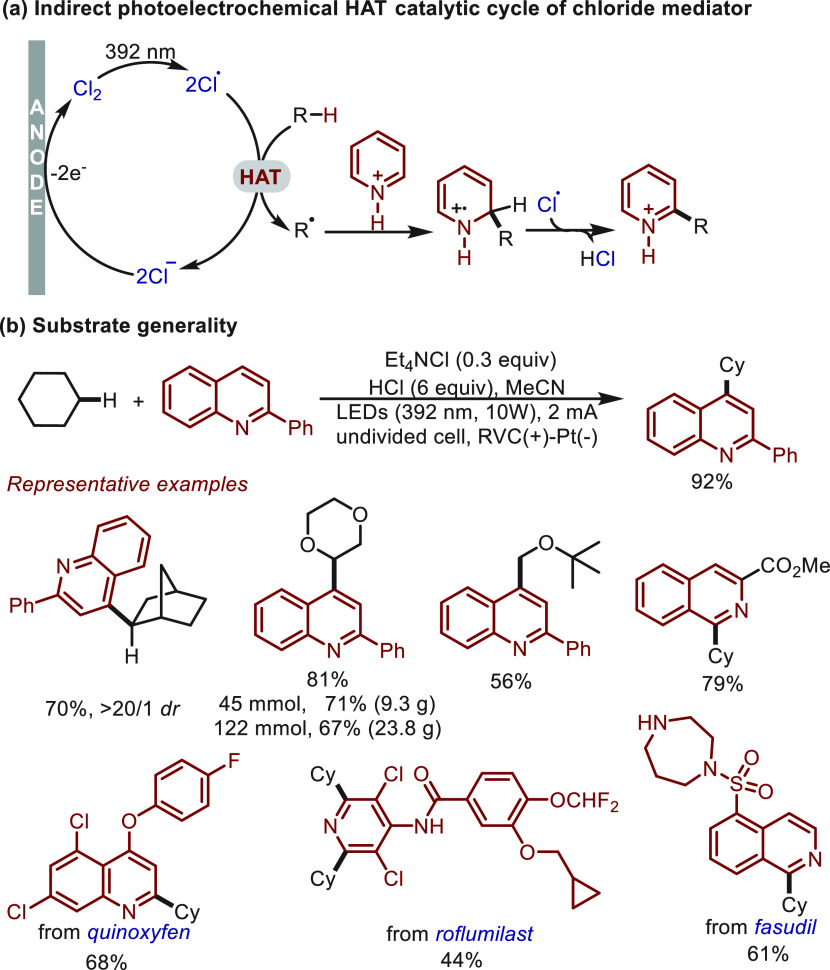
Electrochemical Minisci-Type Reaction Enabled by Indirect HAT Catalysis

In late 2020, a simple chloride anion was employed by Xu^51^ as an effective mediator for a Minisci-type reaction (Scheme 14). Under a purple light (392 nm), chlorine radical was produced from the anodically generated Cl_2_, and it subsequently triggered a HAT process to give nucleophilic C-radicals from alkanes (Scheme 14a). In the presence of acidified heterocycles, cross-coupling products were rapidly generated. Evaluation of the scope of substrate revealed that this transformation tolerated various densely functionalized bioactive molecules (e.g., quinoxyfen, roflumilast, fasudil) in addition to common alkane and heterocycle substrates (Scheme 14b). The practical nature of the photoelectrochemical HAT protocol was also highlighted by its application in a preparative scale reaction with 1,4-dioxane.

**Scheme 15:**
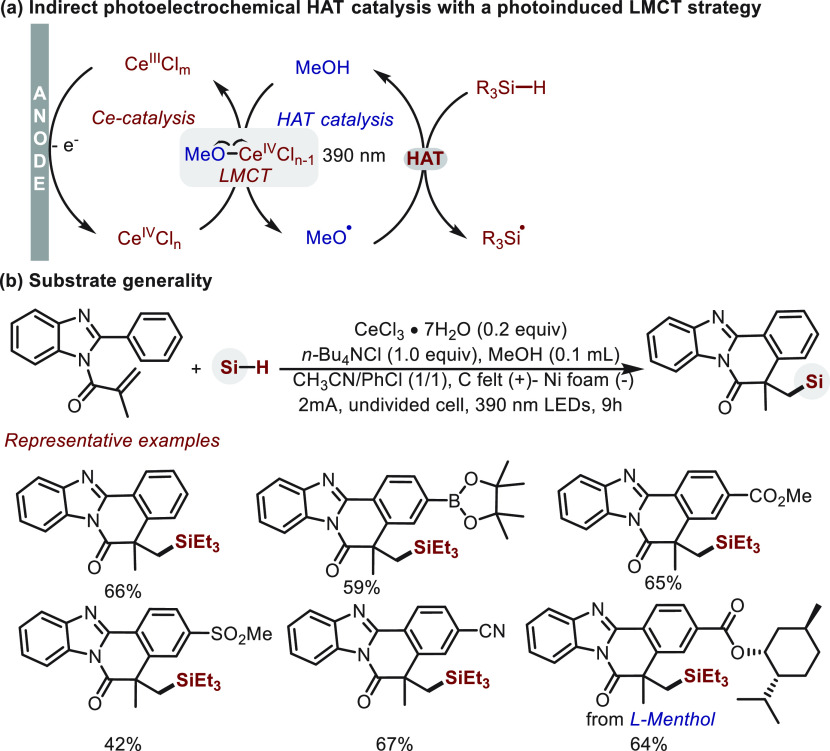
Silyl Radical Generation Enabled by Indirect Photoelectrochemical HAT Catalysis

In the same year, Zeng and Xu^69^ used a strategy of photoinduced LMCT for the activation of Si–H bonds (Scheme 15). Under anodic electrolysis, CeCl_3_ was oxidized to afford a Ce(IV)–OR complex, and subsequent photoinduced LMCT delivers a mild route to MeO^•^, which triggers HAT catalysis (Scheme 15a). To rationalize the observed selectivity for Si–H (BDE ∼ 96 kcal/mol) over α-Si–C–H (BDE ∼ 92 kcal/mol), a polarity-matching effect is invoked that electrophilic MeO^•^ preferentially abstracts the more “hydridic” hydrogen (Si–H). This approach provided a selective and rapid platform for silyl radical generation. In the presence of acrylamide-derived substrates, silyl radical was intercepted and initiated a cyclization to give general access to benzimidazo-fused isoquinolinones (Scheme 15b).

## 5 HAT Catalysis in the Electrochemical Hydrofunctionalization of Alkenes

Metal hydride hydrogen atom transfer (MH HAT)^86,87^ chemistry has emerged as one of the most efficient tools for the donor HAT process in several alkene hydrofunctionalization reactions. However, the conventional approach commonly involves the use of stoichiometric reductants and oxidants that facilitate the process of metal hydride generation and catalyst regeneration, respectively. The use of organic reductants and oxidants in the same flask significantly restricts their practical application due to safety concerns. In this context, Lin,^57^ Baran,^59^ and Zhu^58^ demonstrated electrochemical solutions for this chemistry through anodic oxidation or cathodic reduction processes.

### 5.1 Anodic Hydrofunctionalization of Alkenes by Donor HAT Catalysis

**Scheme 16:**
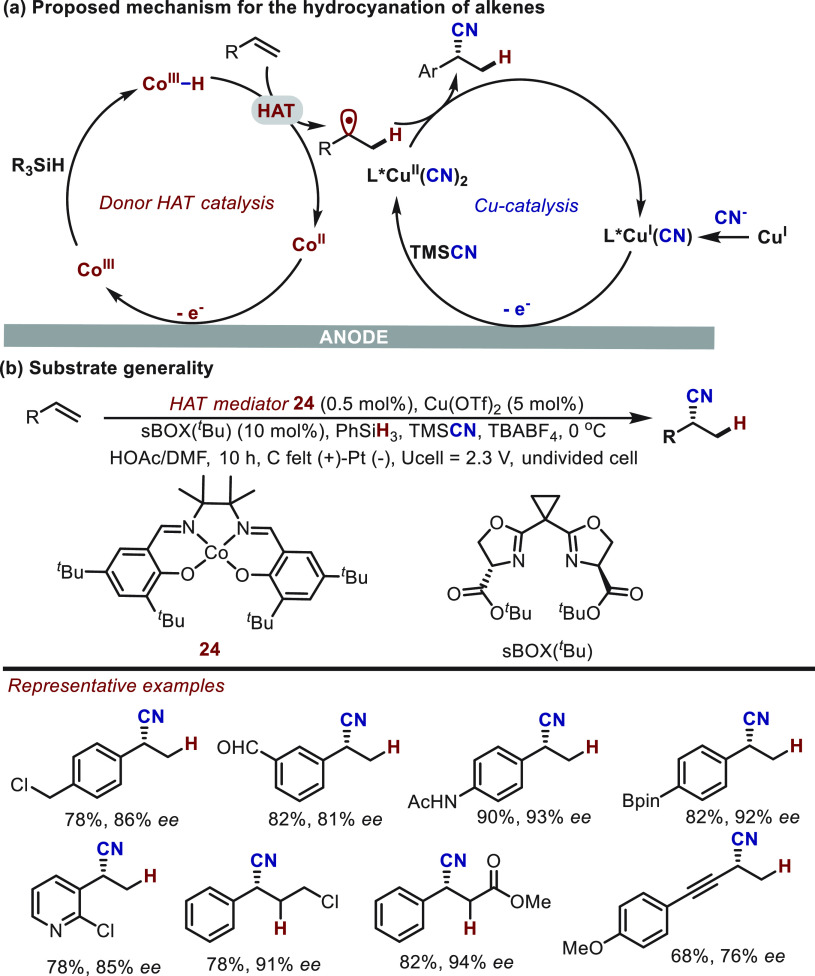
Electrochemical Hydrocyanation of Alkenes Enabled by Donor HAT Catalysis

In 2020, Lin^57^ and co-workers achieved an enantioselective hydrocyanation of conjugated alkenes by merging donor HAT catalysis with copper-promoted radical cyanation (Scheme 16). In the donor HAT catalysis cycle, a Co(III)–H species was anodically generated from a Co(II)–salen precatalyst in the presence of phenylsilane; Co(III)–H served to transfer a hydrogen atom to an alkene substrate generating an allylic or benzylic radical (Scheme 16a). This intermediate subsequently proceeds via a copper-catalyzed cyanation to deliver the chiral nitrile products. The serine-derived bisoxazolines proved to be crucial for the reaction efficiency and stereocontrol. This protocol provides a complementary route to the existing methods for the synthesis of chiral nitriles. For instance, previously challenging substrates like internal alkenes smoothly proceed via hydrocyanation to give the desired nitrile products (Scheme 16b). Moreover, this electrochemical approach showed excellent tolerance for a wide range of sensitive groups such as formyl, amide, boric ester, and pyridyl moieties.

**Scheme 17:**
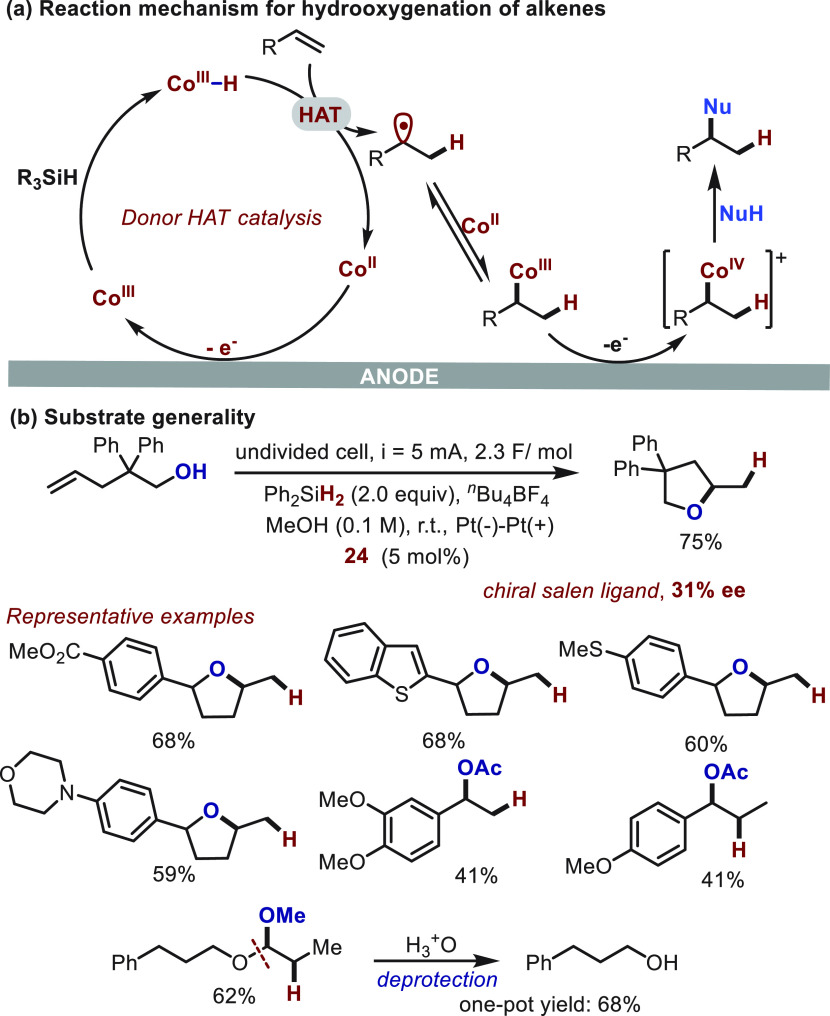
Electrochemical Hydrooxygenation of Alkenes Enabled by Donor HAT Catalysis

Two years later, Zhu^58^ extended the utility of donor HAT catalysis to the hydrooxygenation of alkenes via an interesting Co(II/III/IV) cycle (Scheme 17). Contrary to earlier reports, the alkyl radicals, which are generated *in situ* from alkene substrates, are trapped by Co(II) species to afford an alkyl-cobalt(III), rather than entering the cobalt catalysis cycle (Scheme 17a). Under further anodic oxidation, a highly electrophilic Co(IV) species was produced, and it is primed to react readily with a nucleophile to afford the final product. The putative Co(IV) species was supported by preliminary results of stereocontrol experiments (Scheme 17b). The electrochemical approach shows an appealing functional-group tolerance since the use of typical oxidants was obviated. Some representative products arising from intra- and intermolecular reactions are highlighted below. Additionally, the utility of this protocol was also demonstrated in the deprotection of an allyl group through sequential hydromethoxylation and acidic hydrolysis.

### 5.2 Cathodic Hydrofunctionalization of Alkenes by Donor HAT Catalysis

As described above, anodically donor HAT catalysis still requires stoichiometric silanes as a hydrogen atom source. As concerns related to both sustainability and cost have established themselves at the forefront of modern catalysis, attention has increasingly shifted to using protons (from water and acid) as the hydrogen atom source. The Peters^88,89^ group has designed an elegant cobaltocenium redox mediator to facilitate electroreductive concerted proton–electron transfer (CPET). Employing this novel strategy, electroreductive Pinacol coupling and hydrogenation of fumarate were readily achieved under acidic conditions.

**Scheme 18:**
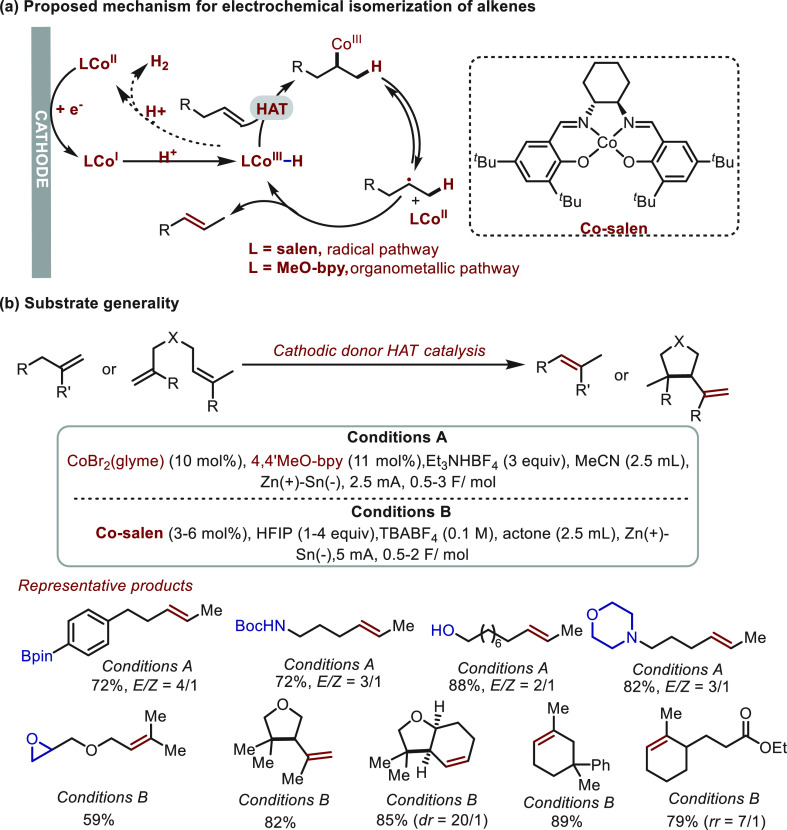
Electrochemical Isomerization of Alkenes

Recently, Baran^59^ and co-workers reported impressive work in cathodic donor HAT catalysis which draws inspiration from the field of energy catalysis (Scheme 18). These authors demonstrated the robustness of the method in alkene isomerization (and semihydrogenation of alkyne, which is outside the scope of this Review). Cathodically generated Co(III)–H species are well-known to promote hydrogen evolution. Through interception with alkenes, Co(III)–H can serve as an efficient hydrogen atom donor and rapidly convert alkene to a Co(III)-alkyl species. This active species can proceed via two distinct pathways to afford final products; the product obtained is controlled by the identity of the ligand coordinated to cobalt (Scheme 18a). Salen ligands promote a weakening of the Co–C bond (20–27 kcal/mol) that resemble diradicals so that Co(III)-alkyl proceeds along a radical pathway to give the alkene isomerization product. In contrast, bipyridine ligands result in stronger Co–C bonds, and an organometallic pathway (β-H elimination) dominates. This methodology is tolerant of a broad range of mono- and disubstituted alkenes and some synthetically useful but sensitive functional groups (e.g., −OH, Bpin, amide, amine, epoxy) (Scheme 18b). The radical pathway enabled by the Co-salen catalyst also provided a general platform for diene cycloisomerization.

**Scheme 19:**
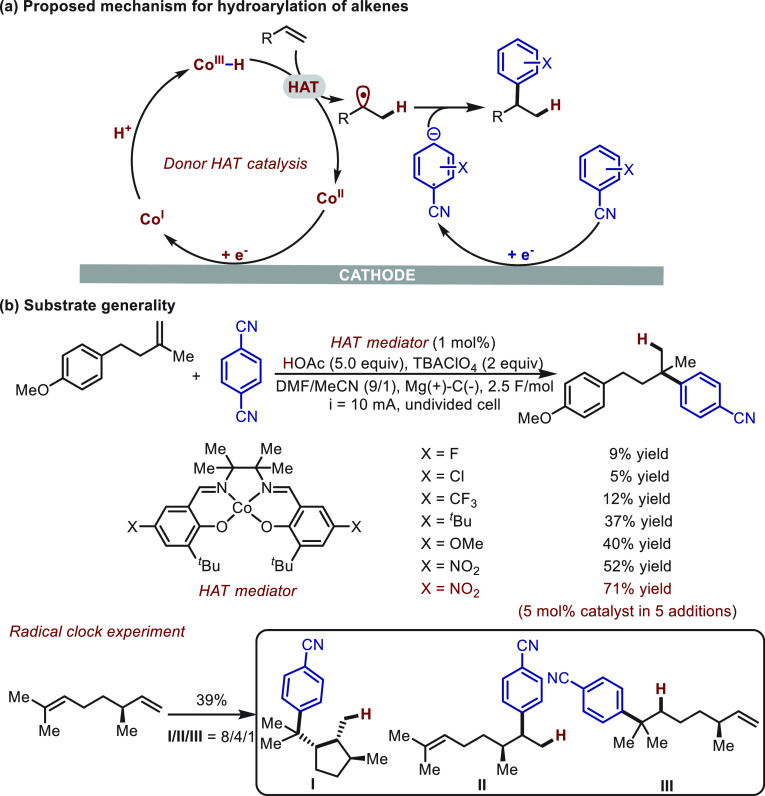
Electrochemical Hydroarylation of Alkenes

Independently, Lin^60^ also demonstrated that cobalt-catalyzed hydrogen evolution reactions (HERs) can be intercepted by a HAT toward alkene hydrofunctionalization (Scheme 19). Using insights obtained from systematic spectroscopic and electroanalytical investigations, Lin arrived at a Co-salen catalyst bearing an electron-donating group as a proposed donor HAT mediator. The nitro-substituted Co-salen catalyst was evaluated as the optimal one, since the nitro group could be reduced *in situ* to an electron-rich group (Scheme 19b). Under cathodic reduction and in the presence of acetic acid, Co(III)–H was generated rapidly, and it subsequently acts as a hydrogen atom donor to alkene substrates triggering the deuteration and hydroarylation of alkenes. The radical mechanism was supported by the detection of “radical” cyclization products when citronellene was employed as a substrate.

## 6 Conclusions and Outlook

### 6.1 Advances

Electrochemically driven HAT catalysis can activate C(sp^3^)–H via hydrogen atom abstraction processes despite large potential differences between substrate and catalyst. Moreover, it also serves as a robust tool for the hydrofunctionalization of alkenes with electrochemically generated metal-hydride species, obviating conventional oxidants and reductants. Consequently, electrochemically driven HAT catalysis provides an appealing platform for both C(sp^3^)–H functionalization and hydrofunctionalization of alkenes featuring high selectivity, excellent functional-group tolerance, and mild reaction conditions.

With these attractive design features, recent years have witnessed tremendous progress in the field. The significant milestones are concluded. Inspired by the seminal work of Grochowski,^71^ NHPI was first introduced to the arena of synthetic electrochemistry by the Masui group^72−74^ in the activation of C(sp^3^)–H bonds. With increasing concerns about sustainable chemistry, tremendous effort has been devoted to synthetic organic electrochemistry in the 21st century. The electrochemically driven HAT catalysis also revives with the promotion of the theoretical study in the HAT and the related study on NHPI. In 2016, Baran^44^ demonstrated the usefulness of Cl_4_NHPI in allylic C(sp^3^)–H oxygenation, and the HAT catalytic mechanism was found to be operational in the transformation. Subsequently, the utility of the NHPI mediator was largely extended to other transformations involving C(sp^3^)–H and Si–H functionalization by the Stahl,^77,78^ He,^80^ and Zhang^79^ groups. Furthermore, direct HAT mediators based on nitrogen-centered radicals were subsequently developed and employed by the Baran,^45,46^ Lei,^47^ Park,^81^ Sun,^68^ and Zhang^70^ groups. In 2020, anodic donor HAT catalysis was reported by Lin and co-workers^57^ for the enantioselective hydrocyanation of alkenes, and this strategy was further expanded to the hydrooxygenation of alkenes by Zhu.^58^ Shortly thereafter, photoredox catalysis and synthetic electrochemistry were combined, and the photoelectrochemical HAT catalysis strategy was developed by the Lambert,^50^ Xu,^51^ and Lei^84^ groups. The fusion of these two methods provides a versatile tool for other transformations as demonstrated in the reports of the Zeng,^69^ Wang,^49^ and Liu^48^ groups. Specifically, Liu^48^ achieved impressive progress in the enantioselective cyanation of benzylic C(sp^3^)–H with the merger of photoelectrochemical HAT catalysis with copper catalysis. In 2022, Baran^59^ and Lin^60^ independently developed the strategy of cathodic donor HAT catalysis, which substantially improves the practicality of donor HAT chemistry as compared with anodic HAT catalysis.

### 6.2 Challenges

Tremendous progress has been achieved in electrochemically driven HAT catalysis. However, it is still in the infancy of its development when compared with the well-established photoinduced HAT catalysis. Two major challenges are involved in electrochemically driven HAT catalysis. The first is that most HAT mediators show low efficiency and inferior tunability. Recently, Stephenson’s mechanistic study^90^ revealed that the oxygen-centered radical PINO undergoes base-assisted decomposition. The presence of side reactions (radical dimerization and decomposition) of the HAT mediators and the short lifetime of the radical species commonly requires high catalyst loading, even stoichiometric “catalysts”. Another handicap is the limited categories of HAT mediators which results in poor tunability and allows only a few choices for a diverse set of desirable transformations. A second challenging issue related to the HAT mediator is that they commonly fail to provide enantioselectivity in the reaction. Although some impressive enantioselectivity has been achieved by virtue of tandem approaches employing copper-catalyzed radical coupling, using a chiral HAT mediator to direct the construction of chiral centers has received far less attention.

### 6.3 Opportunities

Electrochemically driven HAT catalysis provides a versatile and mild platform for both acceptor and donor HAT chemistry. Moreover, the process of electrochemically driven HAT catalysis can be precisely tuned by dialing in both the current and electrode potentials. Consequently, a combination of acceptor HAT chemistry and donor HAT chemistry might be tolerated in an electrochemical system with a convergent paired electrolysis technology, thus providing an electrochemical solution for redox-neutral transformations. Alternatively, the attractive tunability of electrochemically driven HAT catalysis makes it possible to combine HAT catalysis with metal-catalyzed asymmetric radical coupling. We believe that electrochemically driven HAT catalysis would enable an enantioselective platform for radical transformations. Additionally, the incorporation of electrochemistry with photoredox catalysis could provide more diverse choices for HAT catalysts, and the utility of HAT catalysis could be significantly expanded.

In summary, electrochemically driven HAT catalysis has received ever-increasing attention and has already been demonstrated to be capable of the direct functionalization of C(sp^3^)–H bonds and hydrofunctionalization of alkenes. Although there remain some challenging issues associated with the low efficiency of the HAT mediator and the inferior stereocontrol imparted, it does provide promising solutions for asymmetric radical transformation and otherwise challenging transformations via more conventional approaches.
